# *N*-Acetylcysteine Treatment Restores the Protective Effect of Heart Ischemic Postconditioning in a Murine Model in the Early Stages of Atherosclerosis

**DOI:** 10.3390/ph18071014

**Published:** 2025-07-08

**Authors:** Tamara Zaobornyj, Virginia Perez, Georgina Ossani, Tamara Mazo, Eugenia Godoy, Jorge Godoy, Yohana Yanaje, Camila Musci-Ferrari, Mario Contin, Valeria Tripodi, Magali Barchuk, Gabriela Berg, Ricardo J. Gelpi, Martin Donato, Veronica D’Annunzio

**Affiliations:** 1 Instituto de Bioquímica y Medicina Molecular (IBIMOL UBA-CONICET), Facultad de Farmacia y Bioquímica, Universidad de Buenos Aires, Buenos Aires 1113, Argentina; tamaraz@ffyb.uba.ar (T.Z.); camilamuscif@gmail.com (C.M.-F.); 2 Instituto de Fisiopatología Cardiovascular (INFICA), Facultad de Ciencias Médicas, Universidad de Buenos Aires, Buenos Aires 1114, Argentina; mvperez@fmed.uba.ar (V.P.); tamarammazo@gmail.com (T.M.); egn.godoy@gmail.com (E.G.); jorge_godoy@live.com.ar (J.G.); yohana.lilian@gmail.com (Y.Y.); mdonato@fmed.uba.ar (M.D.); 3 Departamento de Patología, Facultad de Medicina, Universidad de Buenos Aires, Buenos Aires 1114, Argentina; georginaossani@gmail.com; 4 Departamento de Tecnología Farmacéutica, Facultad de Farmacia y Bioquímica, Universidad de Buenos Aires, Buenos Aires 1114, Argentina; mcontin@ffyb.uba.ar (M.C.); vtripodi@ffyb.uba.ar (V.T.); 5 Instituto de Fisiopatología y Bioquímica Clínica (INFIBIOC), Departamento de Bioquímica Clínica, Cátedra de Bioquímica Clínica I, Facultad de Farmacia y Bioquímica, Universidad de Buenos Aires, Buenos Aires 1114, Argentina; mbarchuk@docente.ffyb.uba.ar (M.B.); gaberg@ffyb.uba.ar (G.B.)

**Keywords:** ischemia/reperfusion injury, ischemic postconditioning, high-fat diet, redox balance, *N*-acetylcysteine

## Abstract

**Background/Objectives:** Ischemic postconditioning (IP) is a well-established intervention that mitigates this damage by activating endogenous cardioprotective pathways. However, the presence of comorbidities such as dyslipidemia can disrupt these protective mechanisms and abolish the infarct-sparing effect typically induced by IP. In this context, identifying pharmacological strategies to restore cardioprotection is of clinical relevance. This study aimed to evaluate whether *N*-acetylcysteine (NAC), a glutathione precursor with antioxidant properties, can restore the infarct-limiting effect of IP compromised by HFD-induced oxidative stress. **Methods:** Male mice were fed a control diet (CD) or HFD for 12 weeks. NAC (10 mM) was administered in drinking water for 3 weeks before ex vivo myocardial ischemia/reperfusion (I/R) injury (30 min ischemia/60 min reperfusion). In IP groups, six cycles of brief I/R were applied at the onset of reperfusion. Infarct size, ventricular function, redox status (GSH/GSSG), lipid profile, and histology were evaluated. **Results:** NAC improved the lipid profile (HDL/non-HDL ratio) and enhanced the infarct-sparing effect of IP in CD-fed mice. In HFD-fed mice, NAC restored the efficacy of IP, significantly reducing infarct size (HFD-I/R-NAC: 39.7 ± 4.5% vs. HFD-IP-NAC: 26.4 ± 2.0%, *p* < 0.05) without changes in ventricular function. The ratio of oxidized/reduced glutathione (GSSG/GSH) is depicted. Under basal conditions, the hearts of mice fed an HFD exhibited a shift towards a more oxidized state compared to the control diet CD group. In the I/R protocol, a significant shift towards a more oxidized state was observed in both CD and HFD-fed animals. In the IP protocol, the GSSG/GSH ratio revealed a tendency to basal values in comparison to the I/R protocol. The analysis indicates that animals subjected to I/R and IP protocols in conjunction with NAC show a tendency to reach basal values, thus suggesting a potential for the reduction in ROS. **Conclusions:** NAC treatment mitigates oxidative stress and restores the cardioprotective effect of ischemic postconditioning in a model of early-stage atherosclerosis. These findings support NAC as a potential adjunct therapy to improve myocardial resistance to reperfusion injury under dyslipidemic conditions

## 1. Introduction

Cardiovascular disease is a leading cause of mortality worldwide, particularly in developed countries [1]. Myocardial ischemia/reperfusion (I/R) injury represents a clinically relevant problem associated with ischemic heart disease, myocardial infarction, and the implementation of reperfusion therapies. Reperfusion injury encompasses a wide range of pathological alterations, including contractile dysfunction, metabolic disturbances, subcellular structural damage, apoptosis, arrhythmogenesis, and irreversible damage of cardiomyocytes [2]. These detrimental effects are a consequence of various pathological processes triggered during I/R, such as oxidative stress. Therefore, attenuating the progression of I/R-induced oxidative damage in the heart through diverse cardioprotective strategies may offer therapeutic benefit for the heart subjected to I/R injury, especially in patients with comorbidities, such as dyslipidemia [3]. Cardioprotective strategies, which include ischemic postconditioning (IP), are aimed to protect the heart from I/R-induced injury. Although IP has been extensively documented in experimental models, translating these findings into consistent clinical benefits remains a significant challenge [4,5].

A wide range of factors have been implicated in the development of acute myocardial infarction (AMI), including a Western-style diet, sex, tobacco use, diabetes, and dyslipidemia. Among these, dysregulation of lipid metabolism is present in approximately 40% of patients with ischemic heart disease [6] and is considered one of the most influential clinical factors affecting the prognosis of individuals with AMI. Notably, disturbances in lipid metabolism have been shown to promote redox imbalance, thereby contributing to oxidative stress and myocardial injury. Elevated levels of low-density lipoprotein cholesterol (LDL cholesterol) have been shown to promote increased production of reactive oxygen species (ROS). This rise in ROS generation may contribute significantly to the exacerbation of myocardial damage associated with I/R injury [7].

In a previous study, we showed that the cardioprotective effect of IP is abolished in the face of this morbidity, even at the expense of an increase in Thioredoxin-1 (Trx-1) levels. In mice fed a high-fat diet (HFD) for 12 weeks, increased total and non-HDL cholesterol levels were observed, while triglyceride levels and histology remained unchanged, except for mild-to-moderate hepatic steatosis, which did not result in changes in liver enzymes [5]. However, in that previous work, mice fed an HFD exhibited clear signs of endothelial dysfunction. This was evidenced by the response to acetylcholine (Ach) administration in isolated hearts perfused via the Langendorff system. In control diet mice, Ach induced a dose-dependent decrease in coronary perfusion pressure (CPP), consistent with endothelium-dependent vasodilation. However, in HFD-fed mice, Ach administration led to a paradoxical increase in CPP, indicating a vasoconstrictive response, which is a hallmark of early endothelial dysfunction. These findings suggested that the experimental model reflects an early-stage phenotype of atherosclerotic disease, in which a well-established cardioprotective strategy—such as IP—fails to prevent I/R injury. It is of particular interest to investigate the potential of therapeutic interventions to reverse the alterations in IP functionality associated with dyslipidemia, as such findings could have significant clinical implications and guide future research. While animal models have demonstrated the efficacy of exogenous antioxidants, these effects have not been consistently reproduced in clinical settings. This discrepancy is likely attributable to the presence of comorbidities, such as dyslipidemia, in the clinical population [3].

*N*-acetylcysteine (NAC), a cysteine prodrug and glutathione (GSH) precursor, functions as an endogenous antioxidant. Its clinical use as a therapeutic agent has spanned several decades. It has been employed as a mucolytic agent and for the treatment of disorders associated with GSH deficiency [8]. NAC has a well-established redox function and is generally well tolerated, with negligible toxicity even at high doses [9]. Furthermore, it is not influenced by redox imbalance, a characteristic that distinguishes it from other antioxidants, including vitamin E and flavonoids [10]. Indeed, NAC belongs to the extensive category of exogenous antioxidants that have been subjected to rigorous testing to evaluate their cardioprotective potential in the context of cardiac I/R injury. In the context of exogenous antioxidant therapy, NAC merits particular attention due to its specific redox target and lack of pleiotropic effects unrelated to redox modulation. Consequently, NAC—a readily available, cost-effective, and chemically pure antioxidant—has garnered significant attention, as numerous studies have reported its efficacy in mitigating I/R injury [11,12]. However, the potential of NAC administration to restore the cardioprotective effects of IP in a model of early-stage atherosclerosis remains to be investigated.

It is well established that IP reduces the infarct size in healthy experimental models following I/R injury [13,14]. Our previous findings indicated that this cardioprotective effect is abolished in mice fed a high-fat diet, likely due to a redox imbalance [5]. The objective of this study was to determine whether supplementation with the glutathione precursor NAC can restore the cardioprotective effect of IP by reducing infarct size and improving redox balance in a model of early-stage atherosclerosis.

## 2. Results

**Figure 1:**
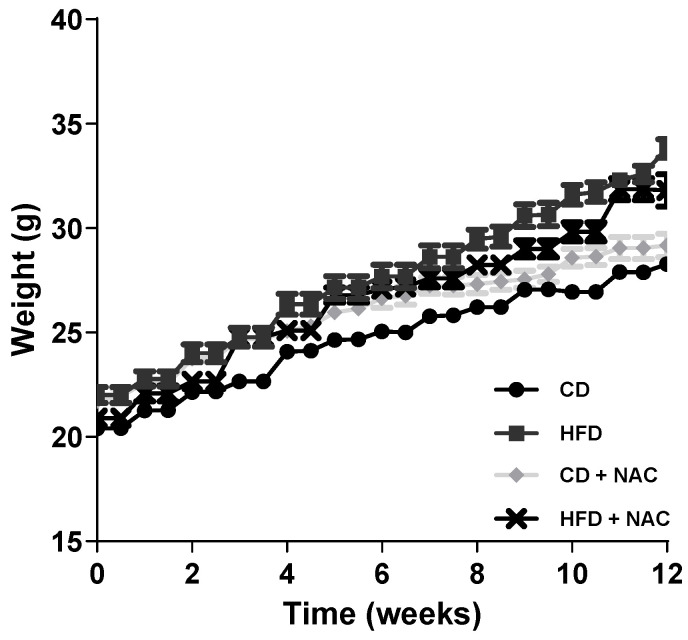
Body weight curve during the 12 weeks of feeding in the group with control diet (CD, *n* = 44), high-fat diet (HFD, *n* = 40), control diet with NAC treatment (CD-NAC, *n* = 42), and high-fat diet with NAC treatment (HFD-NAC, *n* = 42).

During the 12-week duration of the protocol, a weekly evaluation of the body weight of the study groups was conducted. As illustrated in Figure 1, there was an increase in body weight observed among animals subjected to HFD and HFD-NAC, starting from the fourth week of the dietary intervention. Specifically, the body weight of the animals receiving the NAC supplementation (HFD-NAC) was recorded as 30.3 ± 0.3 g, a value that resulted lower than the one of the animals that were fed an HFD, which resulted 31.6 ± 0.3 g.

**Figure 2:**
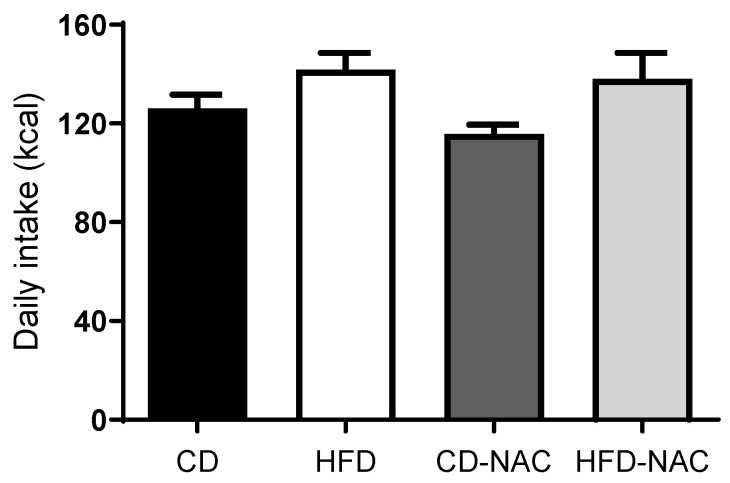
Variation in daily intake during the 12 weeks of feeding in the group with control diet (CD, *n* = 44), high-fat diet (HFD, *n* = 40), control diet with NAC treatment (CD-NAC, *n* = 42), and high-fat diet with NAC treatment (HFD-NAC, *n* = 42).

This weight gain, of approximately 12% compared to the controls, was observed in all groups with HFD and was sustained for 12 weeks, in contrast to those fed a CD and a CD with NAC (CD-NAC) (29.0 ± 0.3 g, 29.2 ± 0.2 g, respectively). Nevertheless, no significant differences were observed among the groups; however, a modest inclination towards diminished body mass was evident in the animals exposed to HFD-NAC. As shown in Figure 2, the daily caloric intake of mice subjected to specific dietary regimens, including CD, HFD, CD-NAC, and HFD-NAC, was examined. The daily intake levels were comparable across all groups (CD: 126 ± 8 kcal, HFD: 142 ± 7 kcal, CD-NAC: 116 ± 5 kcal, and HFD-NAC: 138 ± 11 kcal).

**Figure 3:**
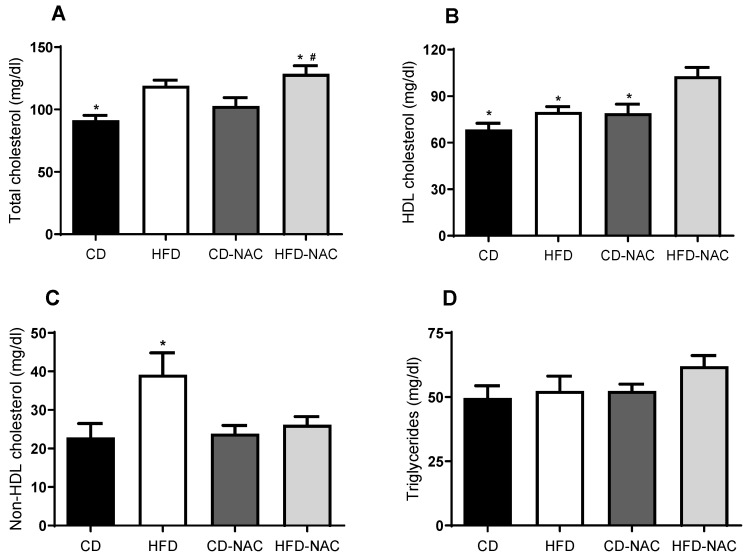
Lipid profile in samples from mice fed with control diet (CD), high-fat diet (HFD), control diet with NAC supplementation (CD-NAC), or high-fat diet with NAC supplementation (HFD-NAC). (**A**) An increase in total cholesterol is observed in those who have an HFD (*n* = 7, * *p* < 0.05 vs. CD; # *p* < 0.05 vs. CD-NAC). (**B**) HDL cholesterol; there is a significant increase in the HFD-NAC group compared to the other groups. (*n* = 7, * *p* < 0.05 vs. HFD-NAC). (**C**) Non-HDL cholesterol; there is a significant increase in HFD with respect to CD (*n* = 7, * *p* < 0.05 vs. CD). (**D**) Triglyceride variation in blood samples; triglyceride levels remained unchanged in the different diet groups (*n* = 7).

Blood samples were collected at the time of euthanasia to determine serum levels of total cholesterol, non-HDL cholesterol, HDL cholesterol, and triglycerides. As illustrated in Figure 3A, the levels of total cholesterol in HFD and HFD-NAC mice increased significantly compared to mice fed a control diet (CD). Specifically, the mean cholesterol levels were 91.3 ± 3 mg/dL in the CD group and 119 ± 3 mg/dL in the HFD group, indicating a substantial increase in cholesterol levels in the former compared to the latter. Similarly, the mean cholesterol levels in the CD-NAC group were 103 ± 7 mg/dL, and the levels in the HFD-NAC group were 129 ± 6 mg/dL, suggesting a comparable increase in cholesterol levels in both groups. Consequently, NAC treatment did not result in alterations in serum cholesterol levels.

As shown in Figure 3, the levels of HDL cholesterol (panel B) and non-HDL cholesterol (panel C) are presented. It has been observed that the treatment of mice with a high-fat diet (HFD) and *N*-acetylcysteine (NAC) resulted in a significant increase in HDL levels (CD: 68.4 ± 3 mg/dL; HFD: 79.9 ± 3 mg/dL; CD-NAC: 79.2 ± 5 mg/dL vs. HFD-NAC: 103 ± 5 mg/dL; Figure 3B). Furthermore, as demonstrated in Figure 3C, a substantial increase in non-HDL cholesterol was observed in mice subjected to an HFD. However, treatment with NAC was associated with a decreasing trend in non-HDL cholesterol levels (CD: 22.9 ± 4 vs. HFD: 39.1 ± 6 mg/dL, CD-NAC: 23.3 ± 2 vs. HFD-NAC: 26.0 ± 2 mg/dL). Finally, Figure 3D clearly shows that there were no changes in triglyceride levels among the different groups (CD: 49.6 ± 4 vs. HFD: 52.4 ± 6 mg/dL; CD-NAC: 52.4 ± 2 vs. HFD-NAC: 62.0 ± 4 mg/dL).

**Figure 4:**
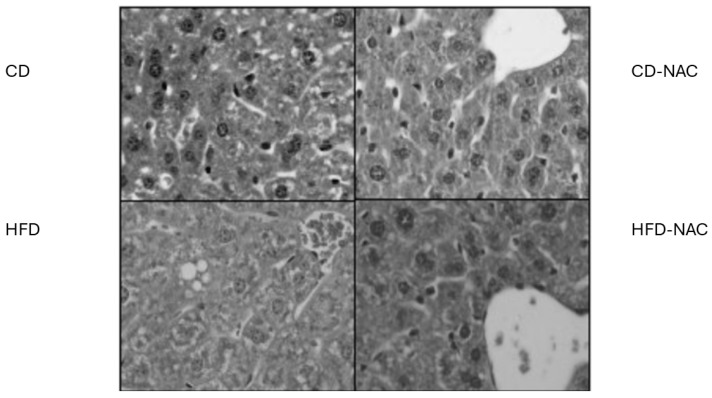
Representative images of liver tissue stained with hematoxylin and eosin in animals fed a CD or an HFD, with or without NAC treatment (×40 magnification) (*n* = 4).

Subsequently, the histological evaluation of liver tissue in animals fed a CD or an HFD, with and without NAC treatment, was conducted (Figure 4). In livers exposed to an HFD, an increase in vacuoles in the interstitium as well as within hepatocytes was observed compared to the group fed a CD. Conversely, in HFD-NAC animals, a decrease in vacuole formation within the tissue was observed. Despite the inability to differentiate between lipids and carbohydrates using hematoxylin and eosin staining, it can be posited that the effect of an HFD on the increase in vacuoles is counteracted by the supplementation with NAC.

Table 1 presents the results of systolic and diastolic ventricular function at baseline and following 30 min of reperfusion in the group of animals subjected to I/R and IP.

In all groups, left ventricular developed pressure (LVDP) and +dP/dt_max_ were measured as an index of contractility. As expected, following a period of prolonged ischemia and subsequent reperfusion, a decline in contractile function was observed, as indicated by a decrease in LVDP and +dP/dt_max_ values in comparison to pre-ischemic baseline levels. The investigation revealed no substantial differences in the recovery of function among the studied groups. Consequently, the implementation of an IP protocol and the administration of NAC did not exert an influence on the observed values. Considering LVEDP as an index of myocardial stiffness and CPP, it was determined that both parameters increased at 30 min of reperfusion as compared to pre-ischemic values. However, no significant differences were observed among the groups. These findings suggest that neither the implementation of the IP protocol nor the administration of NAC resulted in a significant reduction in myocardial stiffness. However, a tendency to decrease in the values of CPP with the application of IP was observed. Considering the aforementioned data, it is evident that neither the dietary interventions nor the administration of NAC exerted any influence on alterations in ventricular function parameters, both prior to and following I/R.

**Figure 5:**
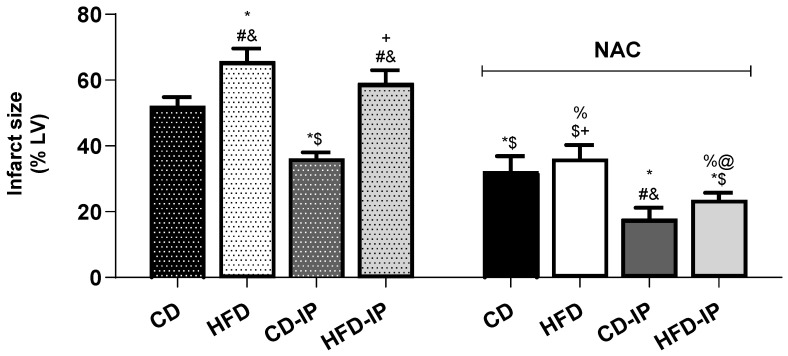
Infarct size was measured as a percentage of total left ventricular area. Infarct size decreased significantly in control animals with IP. The protective effects of IP were abolished in animals fed HFDs. Treatment with NAC recovered the protective effect of IP in HFD animals. All NAC values are lower than their controls. * *p* < 0.05 vs. CD; # *p* < 0.05 vs. CD-IP; $ *p* < 0.05 vs. HFD; & *p* < 0.05 vs. CD-NAC; + *p* < 0.05 vs. CD-IP-NAC; % *p* < 0.05 vs. IP-HFD; @ *p* < 0.05 vs. HFD-NAC. *n* = 7.

Figure 5 presents the infarct size, expressed as a percentage of the LV area, for all the protocols that were studied. Firstly, a 33% reduction in infarct size was observed in the IP group as compared to the I/R group (CD-I/R: 54.4 ± 2% LV vs. CD-IP: 36.6 ± 2% LV). In animals fed an HFD, the I/R group exhibited a larger infarct size compared to the CD-I/R mice (HFD-I/R: 66.4 ± 4% LV); however, in those undergoing an IP protocol, the protective effect was not observed (HFD-I/R: 66.4 ± 4% LV vs. HFD-IP: 62.1 ± 4% LV). In the animals of the IP group treated with NAC, a more pronounced and significant decrease in infarct size was observed compared to the untreated CD-IP group, indicating a synergistic effect of NAC in combination with IP (CD-NAC-IP: 19.1 ± 3% LV vs. CD-IP: 36.6 ± 2% LV). Furthermore, in animals fed an HFD and treated with NAC, a significant decrease in infarct size was observed in both the I/R and IP groups compared to the HFD-I/R and HFD-IP groups (HFD-NAC-I/R: 39.7 ± 4% LV, HFD-NAC-IP: 24.0 ± 2% LV, vs. HFD-I/R: 66.4 ± 4% LV and HFD-IP: 62.1 ± 4% LV). This finding suggests that the protective effect of IP, which was lost in animals fed the HFD, was restored by NAC treatment.

**Figure 6:**
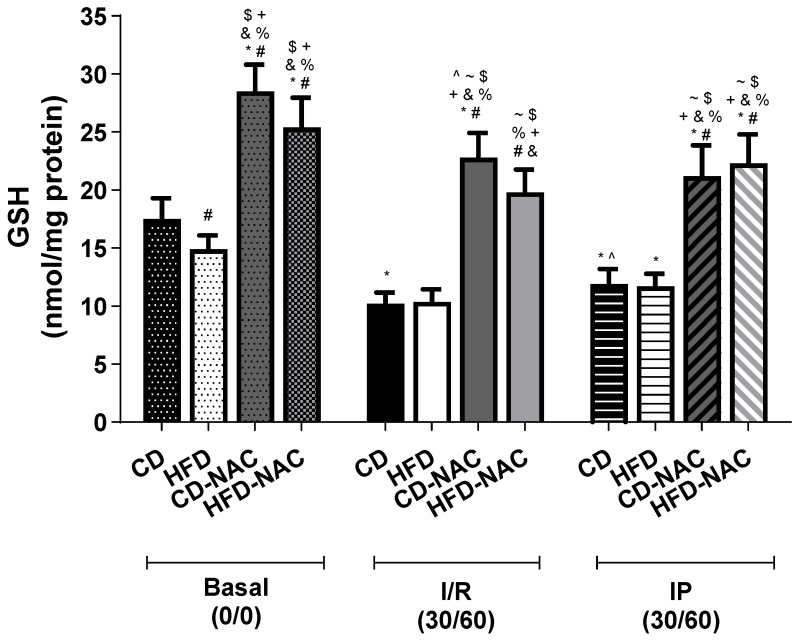
Reduced glutathione (GSH) concentration in the heart. *n* = 7 per group. * *p* < 0.05 vs. CD basal; # *p* < 0.05 vs. CD I/R; $ *p* < 0.05 vs. HFD I/R; & *p* < 0.05 vs. CD IP; + *p* < 0.05 vs. HFD IP: % *p* < 0.05 vs. basal HFD; ~ *p* < 0.05 vs. CD-NAC basal; and ^ *p* < 0.05 vs. CD-NAC basal.

To assess the redox balance of the heart under different experimental conditions, the levels of the reduced (GSH) and oxidized (GSSG) forms of glutathione were examined. As shown in Figure 6, there was a significant increase in cardiac GSH levels in all mice that received NAC supplementation, in comparison to the mice that were not supplemented with NAC. A minimal decline in GSH content was observed in basal HFD compared to their controls, yet no significant alterations were detected in I/R and IP protocols between CD and HFD mice without NAC.

**Figure 7:**
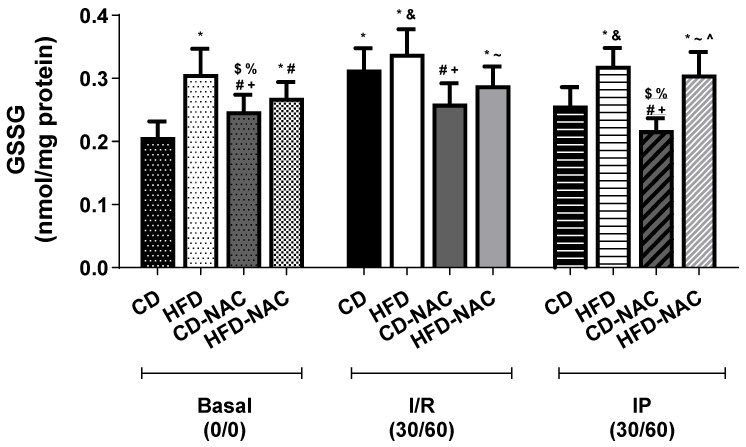
Oxidized glutathione (GSSG) concentration in the heart. *n* = 7 per group. * *p* < 0.05 vs. CD basal; # *p* < 0.05 vs. HFD I/R; $ *p* < 0.05 vs. CD I/R; & *p* < 0.05 vs. CD IP; + *p* < 0.05 vs. I/R HFD: % *p* < 0.05 vs. basal HFD; ~ *p* < 0.05 vs. CD-NAC IP; and ^ *p* < 0.05 vs. CD-NAC basal.

As illustrated in Figure 7, there was a significant increase in GSSG levels in CD-I/R with respect to basal CD in the heart. A slight increase was also observed in mice fed a high-fat diet (HFD) compared to their controls. No significant alterations were observed among HFD groups in the study. In the end, there were no significant changes the various CD-NAC and HFD-NAC protocols.

**Figure 8:**
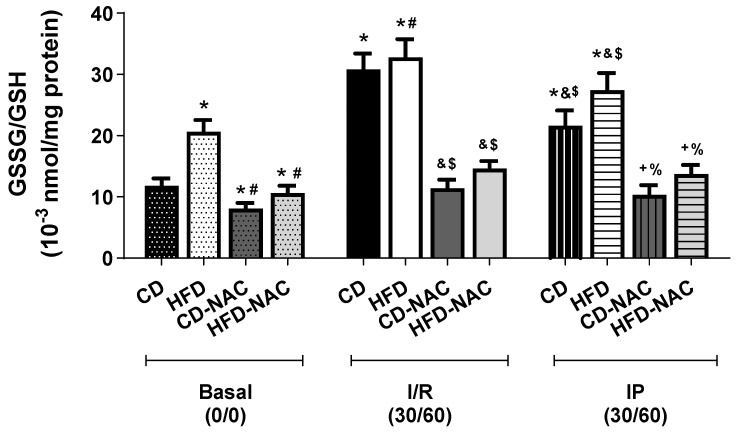
Oxidized/reduced glutathione ratio (GSSG/GSH). The hearts of animals fed HFD were observed to change to a more oxidized state. However, this change to an oxidized intracellular environment returned to pre-ischemic values after the IP protocol in CD groups but not in the IP-HFD group. Regarding those treated with NAC, there is evidence of a significant descent to a pre-ischemic oxidized state. *n* = 7 per group. * *p* < 0.05 vs. CD basal; # *p* < 0.05 vs. HFD I/R; $ *p* < 0.05 vs. CD I/R; & *p* < 0.05 vs. CD IP; + *p* < 0.05 vs. I/R HFD: % *p* < 0.05 vs. basal HFD.

In Figure 8, the ratio of oxidized/reduced glutathione (GSSG/GSH) is depicted. Under basal conditions, the hearts of mice fed an HFD exhibited a shift towards a more oxidized state compared to the control diet CD group. In the I/R protocol, a significant shift towards a more oxidized state was observed in both CD- and HFD-fed animals. In the IP protocol, the GSSG/GSH ratio revealed a tendency to basal values in comparison to the I/R protocol. The analysis indicates that animals subjected to I/R and IP protocols in conjunction with NAC show a tendency to reach basal values, thus suggesting a potential for the reduction in ROS.

## 3. Discussion

The present study demonstrated that NAC treatment effectively restores the cardioprotective effect of ischemic IP compromised by high-fat diet-induced oxidative stress. NAC improved lipid profile and redox balance, reducing infarct size in both control and HFD-fed mice. While ventricular function remained unchanged, the normalization of the GSSG/GSH ratio suggests reduced oxidative damage. These results highlight the therapeutic potential of NAC in enhancing myocardial resilience under dyslipidemic conditions. Overall, NAC represents a promising adjunct strategy for cardioprotection in the presence of metabolic comorbidities.

The findings presented in this study are preceded by another study in which it was shown that despite the absence of significant histological changes in coronary vessels or the aorta, mice fed a high-fat diet for 12 weeks exhibited clear signs of endothelial dysfunction. This was evidenced by the response to acetylcholine (Ach) administration in isolated hearts perfused via the Langendorff system. In control-diet mice, Ach induced a dose-dependent decrease in coronary perfusion pressure (CPP), consistent with endothelium-dependent vasodilation. However, in HFD-fed mice, Ach administration led to a paradoxical increase in CPP, indicating a vasoconstrictive response, a hallmark of early endothelial dysfunction.

This paradoxical vasoconstrictive effect of acetylcholine has been widely recognized as a surrogate marker of endothelial dysfunction, even in the absence of structural vascular changes. Classic studies, such as those by Ludmer et al. [15] and Egashira et al. [16], demonstrated that this phenomenon corresponds to early stages of atherosclerosis and reflects impaired nitric oxide bioavailability in the vascular endothelium. Therefore, our current findings, together with this previously reported evidence, support the hypothesis that NAC supplementation may serve as a preventive nutraceutical strategy to protect endothelial function, enhance cardioprotective mechanisms such as ischemic postconditioning, and ultimately reduce myocardial injury and its long-term consequences, including adverse remodeling and progression to heart failure.

Feeding mice with an HFD for 12 weeks resulted in a phenotype that was indicative of the initial stages of atherosclerosis. This was characterized by higher levels of cholesterol and triglycerides in comparison to mice that were fed a CD. Conversely, the administration of NAC resulted in an augmentation of HDL cholesterol levels, while total cholesterol levels remained consistent with those observed in the CD and HFD groups. Secondly, it was observed that high-fat diet had an influence on infarct size and myocardial protection conferred by ischemic postconditioning, which was abolished by HFD. This was evidenced by the fact that there was a greater infarct size in the HFD-fed mice than in the CD-fed mice subjected to an I/R protocol. Furthermore, the IP intervention resulted in a reduction in infarct size in the CD group but not in the HFD group. In the context of the present study, the administration of NAC in both the CD and HFD groups resulted in an important reduction in infarct size, a phenomenon that was also observed in the IP groups. The present findings suggest that NAC not only protects mice with CD and HFD from I/R injury, but also enhances the protective effect of IP, even in mice fed with HFD.

Conversely, when assessing ventricular function, no significant differences were observed among the various groups studied following I/R and IP, nor with NAC treatment. With respect to glutathione levels, the hearts of mice fed an HFD exhibited a more oxidized redox state compared to the CD group under basal conditions. Moreover, the I/R procedure significantly increased oxidized glutathione levels in both groups. When treated with NAC, this effect was reversed, approaching a value close to the preischemic state. This shift in value is likely to influence the reduction of infarct size.

Other authors showed that mice fed a high-fat diet exhibit greater metabolic, inflammatory, and oxidative stress alterations compared to other strains, such as FVB [17,18]. For these reasons, we chose the C57BL/6 strain. The results of the experiment demonstrated that the mice in the HFD group showed a higher weight compared to the mice in the CD group. This difference in weight was not mitigated by the supplementation with NAC. It is also important to mention that the daily caloric intake did not suffer significant variations among groups. Our findings are consistent with the results of other researchers, who did not observe significant variations in caloric intake upon treatment with NAC [19,20]. This suggests that this compound does not cause alterations in the appetite of mice.

This study showed a significant increase in total cholesterol levels in mice fed a high-fat diet (HFD). Similar findings were reported by Nie et al. [21], although their diet composition and duration differed (12% lard, 65% fat, for 6–8 weeks). NAC supplementation did not significantly affect cholesterol levels, as values in the HFD and HFD-NAC groups were comparable to their respective controls. Contrastingly, Lin et al. [20] reported decreased cholesterol levels with NAC in Balb/cA mice on a 21.5% fat diet for 4 weeks. Another study observed similar effects in rats on a 55% fat diet with NAC, also over 4 weeks [18]. Korou et al. [22], using C57BL/6 mice and a high-cholesterol diet (2% cholesterol, 0.5% cholic acid) for 8 weeks, noted a non-significant reduction in cholesterol with NAC. These discrepancies may be due to differences in diet composition, treatment duration, and animal models.

Notably, NAC treatment altered cholesterol distribution, with the HFD-NAC group showing a significant increase in HDL cholesterol and a decreasing trend in non-HDL cholesterol. Similar outcomes were reported in Wistar rats on a sucrose-rich diet and in models of non-alcoholic fatty liver disease under HFD, where NAC increased HDL and reduced non-HDL cholesterol levels [23,24]. These improvements may be attributed to antioxidant properties of NAC, which reduce oxidative stress and lipoprotein peroxidation, promoting lipid clearance—particularly of non-HDL particles—and enhancing HDL levels [25]. This shift in the HDL/non-HDL ratio could lower the risk of ischemic heart disease.

Histological analyses have shown that an HFD induces hepatocyte vacuolization, likely due to lipid accumulation and steatosis, a pattern not seen in CD-fed mice [5]. Similar signs of steatosis and hepatic distention were observed in cholesterol-rich diets [22] and after prolonged HFD exposure in C57BL/6 J mice [26]. Ma et al. [27] also reported marked vacuolization with HFD. NAC treatment appears to reverse these effects, reducing vacuolization [28], lipid droplet size [29], and hepatic triglyceride/cholesterol levels [27], suggesting its potential to prevent steatosis development under HFD conditions. Taking this evidence into account, the administration of NAC in conjunction with a high-fat diet has been shown to prevent the vacuolation of hepatocytes. This, in turn, would reduce the accumulation of lipids in liver tissue, thereby mitigating the risk of developing steatosis.In the results obtained, after the 30 min of ischemia, there was an expected reduction in the contractile state during reperfusion in all groups. This was assessed with the decrease in LVDP and +dP/dt_max_. Added to this was the increase in myocardial stiffness (LVEDP) at reperfusion. A slight post-ischemia increase in coronary vascular resistance (CPP) was also observed during reperfusion, but without significant values among all groups studied. This means that IP had no effect on the recovery of ventricular function. On one hand, it should be considered that the perfusion in the Langendorff system is retrograde, that there is no isolated ejection of the heart, in addition to the fact that it is isovolumic, which maintains the loading conditions constantly to evaluate the contractile state. The heart rate is also kept constant, and all these factors are the ones that mainly affect ventricular function. On the other hand, the lack of improvement in the recovery of ventricular function is due to post-ischemia ventricular dysfunction (myocardial stunning), which is reversible if there is no necrosis. There are areas of stunned myocardium that are in peripheral areas to the infarction zones, which are associated with the phenomenon of I/R injury [30]. Unfortunately, with our experimental model, we cannot evidence the improvement of ventricular function, since the follow-up can only be 2 h. It should be noted that there are several studies in which there were improvements in infarct size with different protection strategies but without improvements in ventricular function [4,31,32,33,34]. In this context, it is plausible to hypothesize the presence of post-ischemic ventricular dysfunction (stunned myocardium), which has been shown to reverse following approximately 72 h of reperfusion. Consequently, alterations in infarct size, as determined 120 min following reperfusion, would not substantially impact the enhanced recovery of ventricular function [30] in the acute phase, as evidenced by our model.

In the HFD-fed mice, there is a significant increase in infarct size after I/R. However, the application of an IP protocol did not reduce infarct size in these mice as in their counterpart (CD). There are several studies that show that protective effects of IP are abolished in the presence of hyperlipidemia [4,35,36,37]. On the other hand, with NAC treatment, a significant decrease in infarct size was evidenced in all groups. Particularly, in mice fed with an HFD, the IP protocol achieved a larger reduction in infarct size. NAC has a myocardial protective effect that prevents I/R damage in ex vivo models of isolated heart and in vivo models of heart infarction [38,39], which promotes the restoration of the protective effect of IP. Regarding treatment with NAC, a study with male Wistar albino rats [40] pretreated with NAC showed a significant decrease in infarct size. The same occurred in other Wistar rats pretreated with NAC for seven consecutive days by tube before myocardial injury induced by the I/R protocol [41]. Also, a group of Wistar rats treated with NAC 5 min before occlusion with a dose of 15 mg/kg [38] presented a decrease in infarct size. In addition, in patients with AMI, the effects of NAC at high intravenous doses with a low dose of background nitroglycerine were evaluated [42], where a decrease in infarct size was also observed. Based on studies carried out with a high-fat diet and NAC treatment, it can be concluded that feeding with an HFD not only increases infarct size compared to I/R but also counteracts the protective effect of IP, but this is reversed by NAC supplementation, probably due to its antilipidemic and antioxidant effects. The originality of our work lies in demonstrating a protective effect of NAC on infarct size in an HFD model. Unlike previous studies that primarily focused on metabolic or hepatic outcomes, our findings reveal that NAC restores the infarct-sparing effect of ischemic postconditioning under dyslipidemic conditions, highlighting its potential as a cardioprotective strategy in the context of diet-induced oxidative stress.

The contribution of ROS generation to I/R injury has prompted several studies exploring the efficacy of antioxidant therapies, including some clinical trials. The outcomes have been inconclusive, which is not unexpected given that ROS production can be advantageous as a signaling mechanism and in cardiac protection via preconditioning interventions [43]. Nevertheless, it has been established that exogenous antioxidant and the administration of interventions which upregulate transcription factors involved in redox homeostasis, as well as vitamins (A, C and E), have been shown to exert beneficial effects against I/R. These latter observations demonstrate the cardioprotective effects of various interventions in I/R hearts, attributable to their antioxidant activities [16,44].

In the results obtained, the increase in GSH was significant in the groups treated with NAC compared to their control groups. Similar results were obtained in the study of Ommati et al. [45] in bile duct ligated mice, which showed high GSH levels compared to their controls without NAC. In another study with C57BL/6 J mice, an increase in GSH levels was also obtained [46], with the difference that NAC was co-administered with glycine. This confirms that NAC helps to restore GSH levels, as it is rapidly converted to GSH [47], causing its increase. On the other hand, GSSG levels increase significantly after I/R and slightly in HFD-fed animals. This can be attributed to the fact that an HFD promotes dyslipidemia, which in turn leads to redox imbalance [48]. There was no significant improvement with the application of IP, so treatment with NAC did not help to reduce GSSG levels, unlike GSH. Based on the results obtained from GSH and GSSG levels, a GSSG/GSH ratio was calculated and showed a more oxidized state in HFD-fed mice, especially after I/R, so that the HFD had a negative effect on the redox state, creating an imbalance. When the IP protocol was applied, the GSSG/GSH ratio was not restored, so the protective effect of IP was not sufficient to reduce the redox imbalance. However, after supplementation with NAC, a decrease in the oxidized state was clearly demonstrated, especially in the HFD groups treated with NAC (HFD-NAC). This is related to the increase in GSH induced by NAC, which benefits the system by bringing it to a less oxidized state, which attenuates oxidative stress in the HFD, reducing the effects of I/R and even restoring the protective effect of IP. Accordingly, there are studies that confirm that the use of NAC reduces oxidative stress. For example, Paschalis et al. [49] showed that NAC supplementation in patients with low levels of GSH and sedentary lifestyle, there is a reduction in oxidative stress. Similar studies have demonstrated a reduction in oxidative status [50,51].

Changes in the intracellular balance between GSH and GSSG play a significant role in determining the redox status and signaling of a cell. Plasma GSH redox in humans becomes oxidized with age, in response to oxidative stress (chemotherapy, smoking), and in common diseases (type 2 diabetes, cardiovascular disease) [52]. In this study, two factors have been shown to induce a shift towards the oxidized form of GSH: I/R and chronic HFD. As has been shown by us and others [5,53], redox balance of cardiomyocytes plays a key role in the effectiveness of cardioprotective protocols. In this study, NAC supplementation was shown to restore the GSSG/GSH ratio to normal values, providing an adequate redox environment for IP to activate the protective events that afford a reduction in infarct size. Overall, our findings highlight the crucial role of NAC in mitigating oxidative stress by restoring GSH levels and reducing the oxidized state, particularly in HFD-fed mice subjected to I/R. While IP alone failed to counteract the redox imbalance induced by HFD, NAC supplementation effectively decreased the GSSG/GSH ratio, suggesting its potential as an adjuvant strategy to enhance cardioprotection.

Ischemic postconditioning is known to induce the production of several autacoids, such as acetylcholine and adenosine, by cardiomyocytes. These latter activate various signaling pathways that sustain a cardioprotective signal to the mitochondria [54]. In these organelles, mild levels of reactive oxygen species (ROS) are produced. These concentrations of ROS can induce the activation of protein kinases such as Akt, Erk1/2, protein kinase C, and tyrosine kinase, thus providing the adaptive stimulus that will be protective in the subsequent reperfusion insult. It is known that preservation of mitochondrial function with attenuated ROS production, less calcium overload, and inhibition of mitochondrial permeability transition pore opening may provide the protective effect [55]. Intermittent reperfusion induced by IP has also been shown to delay restoration of intracellular pH after reoxygenation by reperfusion, which might contribute to the MPTP inhibition observed in postconditioned hearts [56]. In this scenario, the maintenance of an adequate redox balance, which would allow these signaling pathways to be adequately activated and to induce the functional effects that protect the cardiomyocytes upon reperfusion, seems a potential way for NAC to exert its effects.

These results further strengthen the evidence supporting NAC as a therapeutic agent in conditions characterized by oxidative stress, particularly in the context of metabolic disorders such as dyslipidemia that impair endogenous cardioprotective pathways. Our study adopts a preventive approach, showing that NAC not only improves redox balance but also restores the infarct-sparing effect of ischemic postconditioning and significantly reduces infarct size in a model of early-stage atherosclerosis. These findings suggest that NAC supplementation, when initiated prior to an ischemic event, could effectively limit myocardial damage and positively influence clinical outcomes following infarction. Moreover, by preserving viable myocardium during acute myocardial infarction, NAC may help mitigate adverse cardiac remodeling and delay or prevent progression to heart failure—an increasingly important target in the management of cardiovascular disease. Altogether, this supports the potential use of NAC as a preventive nutraceutical strategy in individuals at high cardiovascular risk, especially those with underlying metabolic comorbidities.

## 4. Materials and Methods

### 4.1. Animal Care

**Figure 9:**
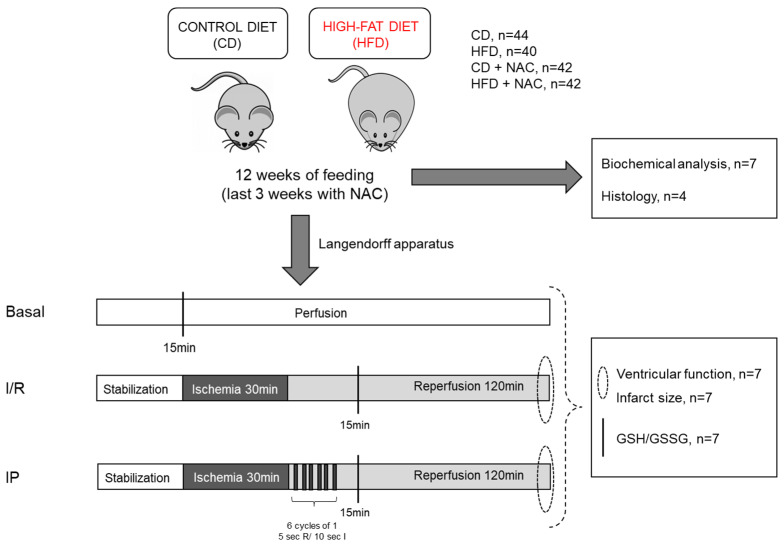
Experimental design.

The animal study protocol was in accordance with the ethical standards of the Animal Care and Research Committee of the University of Buenos Aires (CICUAL UBA RESCD-2023-1423-UBA-DCT#FMED) and the International Guiding Principles for Biomedical Research Involving Animals, as issued by the Council for the International Organizations of Medical Sciences. Male mice of the C57/BL6 strain, with a body weight range of 20–22 g, were used for this study. During the 12-week feeding protocol (Figure 9), mice were provided with water ad libitum and maintained under a 12 h light/dark cycle and controlled temperature (20–22 °C). This was a randomized, double-blind experimental design. The mice were separated into 2 groups according to the diet they were fed with: a control diet (CD) group, which consumed 27.6% of total calories from protein, 13.5% from fat, and 58.8% from carbohydrates; and a high-fat diet (HFD) group, which consumed 18.9% of total calories from protein, 40.7% from fat, and 40.3% from carbohydrates. The diet was formulated by referencing the commercially available diet D12451 from “Research Diets”, which contains 45% fat and 35% carbohydrates. This latter diet was modified by substituting pork fat with high oleic oil and lard. A minor discrepancy was observed in the percentage of fat and carbohydrates. The diet employed in this study exhibited a reduced fat content of 40%, while the carbohydrate content was augmented to 40%.

In the final 3 weeks of the feeding period, a subgroup of the animals was treated with NAC, administered via drinking water at a dose of 10 mM. Some of the studies unraveling the effects of NAC in I/R used doses of 1.5 g/kg/day, which for a human adult would be about 75 g/day. In our study, we considered a water intake of about 6 mL, which means a dose of 0.06 mmol NAC or 9.8 mg mmol/day. The body weight and daily caloric intake of all groups were monitored throughout the feeding period. The total number of mice that received each diet and/or supplementation is indicated in Figure 9.

### 4.2. Experimental Protocols

In the I/R protocol, myocardial infarction was induced with 30 min of global no-flow ischemia, in which total coronary flow provided by the perfusion pump was abruptly stopped. The setting up of ischemia was followed by 120 min of reperfusion. In the ischemic postconditioning protocol (IP), mice were subjected to the I/R protocol. However, at the onset of reperfusion, 6 cycles of 10 s of reperfusion followed by 10 s of ischemia were performed. The complete IP protocol was completed within a span of 2 min. The reperfusion procedure was conducted for a duration of 120 min. The sample size was determined using the G*Power software version 3.1.9.7., as previously described [5].

### 4.3. Isolated Mouse Heart Perfusion Protocol

Immediately after the 12-week feeding protocol, mice were euthanatized and hearts were isolated and perfused using the Langendorff technique, as previously described [5]. Briefly, mice were anesthetized with an intraperitoneal injection of sodium pentobarbital (150 mg/kg) and sodium heparin (500 IU/kg bow, i.p). The heart was then perfused with Krebs bicarbonate-buffered solution (118.5 mM NaCl, 4.7 mM KCl, 24.8 mM NaHCO_3_, 1.2 mM KH_2_PO_4_, 1.2 mM MgSO_4_, 1.5 mM CaCl_2_ and 10 mM glucose). A pacemaker (QRS Ingenieria Médica, La Plata, Argentina) stabilized the heart rate at 471.1 ± 28.2 beats/min. Coronary perfusion pressure (CPP) was recorded with a pressure transducer (Argon Medical Devices, Schiphol-Rijk, The Netherlands) connected to the perfusion line. The heart was perfused at a constant flow of 4.2 ± 0.1 mL/min.

### 4.4. Ventricular Function

Left ventricular developed pressure (LVDP) and the maximal rate of rise of left ventricular pressure (+dP/dt_max_) were determined as contractile state indexes. Left ventricular end-diastolic pressure (LVEDP), which is a myocardial stiffness index, was also measured. The number of animals from each group used in this evaluation was 7.

### 4.5. Infarct Size Measurement

The assessment of the infarct size was performed using 2,3,5-triphenyltetrazolium (TTC, Sigma Aldrich staining, St. Louis, MO, USA) [57]. Following a 120 min reperfusion period, the hearts were subjected to freezing and slicing at a thickness of 1 mm, traversing the entire length from the apex to the base. Sections were subjected to an initial incubation of 20 min in 1% TTC (pH 7.4, 37 °C), followed by immersion in 10% formalin. The implementation of this technique enabled the differentiation of viable sections, which were subsequently stained red, from non-viable sections corresponding to the infarct area. Sections were traced to acetate sheets and planimetered (Image Pro Plus 4.5). Infarct size was expressed as a percentage of the left ventricular area (*n* = 7 per group).

### 4.6. Biochemical Analyses

Blood samples were collected from CD or HFD animals after 12 weeks of exposure and subsequently subjected at 1500× *g* for 15 min. Serum was stored at a temperature of 4 °C for 48 h. The assessment of total cholesterol and triglycerides was conducted using commercial enzymatic kits (Roche Diagnostics GmbH, Mannheim, Germany) within a Co-bas C-501 autoanalyzer, following the methodologies established by the International Federation of Clinical Chemistry (IFCC). The determination of HDL cholesterol was executed through the implementation of a standardized selective precipitation method, employing phosphotungstic acid/MgCl_2_ as precipitating reagent [5]. No HDL cholesterol was determined by calculating the difference between total cholesterol and HDL cholesterol, thereby estimating atherogenic lipoprotein levels. The number of animals from each group used in these experiments was 7.

### 4.7. Histological Analysis

Following the conclusion of the 12-week feeding protocols, mice from each group (*n* = 4, each) were euthanized, and the liver was extracted using microdissection techniques. The organs were fixed in 10% buffered formalin and subsequently embedded in paraffin. To facilitate the analysis of histopathological alterations, 3 mm-thick sections were prepared and subsequently stained with hematoxylin and eosin, employing conventional protocols [5]. Regions of interest were examined on a Nikon microscope and photographed with a digital camera (Melville, NY, USA).

### 4.8. Reduced (GSH) and Oxidized (GSSG) Glutathione Levels

Homogenate (30 µL) were supplemented with 60 µL of cold TFA 10% *v*/*v* with EDTA 1 mM. The samples were maintained on ice for 10 min, then subjected to centrifugation at 9000× *g* for 20 min at 4 °C. Prior to injection, samples were diluted to a ratio of 1/4 with 0.1% *v*/*v* formic acid. The assessment of GSH and GSSG was performed through a micro HPLC-MS/MS method, as previously detailed [58]. The TSQ Quantum Access Max system (Thermo Fisher Scientific, San Jose, CA, USA) was utilized in this method, equipped with a BDS HYPERSIL C18 (Thermo Fisher Scientific, San Jose, CA, USA) column measuring 100 mm × 2.1 mm × 2.4 mm, accompanied by a C18 guard column. Operation was conducted in ESI negative mode. The number of animals from each group used in these experiments was 7.

### 4.9. Statistical Analysis

Data were expressed as mean ± standard error of the mean (SEM). Inter-group comparisons were performed using analysis of variance. The Bonferroni method was used for multiple comparisons corrections. *p* < 0.05 was considered statistically significant.

## 5. Conclusions

This study showed that chronic NAC treatment was able to restore the protective effect of IP, which was lost in mice fed with an HFD. The supplementation of NAC to mice fed with an HFD was associated with a clear change in the proportion of HDL and non-HDL cholesterol, favoring an increase in HDL and a decrease in non-HDL cholesterol. These effects were accompanied by the restoration of GSH levels and a decrease in GSSG levels, which favored the less-oxidized cellular redox state. Not only did GSH levels recover, but they surpassed baseline levels. Therefore, the improvement of the redox balance may restore the cardioprotective effect of IP and, therefore, reduce the infarct size. This work presents new perspectives for the future, as it proposes possible treatments for I/R injury in those individuals who are subject to different comorbidities such as dyslipidemia. In addition, it opens a new window to the study of signaling mechanisms.

## Figures and Tables

**Table 1:** Ventricular function in animals fed a control diet or high-fat diet.

	Groups	Basal	30 min R
LVDP (mmHg)	CD-I/R	96.3 ± 1.0	20.3 ± 3.4 *
	CD-IP	81.3 ± 5.9	19.9 ± 4.2 *
	HFD-/R	98.3 ± 4.5	19.6 ± 2.8 *
	HFD-IP	93.8 ± 11	20.2 ± 3.8 *
	CD-I/R-NAC	97.3 ± 2.0	21.2 ± 4.3 *
	CD-IP-NAC	98.3 ± 6.80	20.5 ± 5.4 *
	HFD-I/R-NAC	76.2 ± 3.53	20.3 ± 3.2 *
	HFD-IP-NAC	80.3 ± 7.78	19.9 ± 3.7 *
LVEDP (mmHg)	CD-I/R	7.3 ± 2.6	37.3 ± 6.8 *
	CD-IP	9.4 ± 1.9	41.9 ± 6.9 *
	HFD-I/R	9.2 ± 6.2	46.4 ± 12.4 *
	HFD-IP	7.7 ± 2.2	46.4 ± 3.1 *
	CD-I/R-NAC	8.6 ± 4.6	42.6 ± 7.6 *
	CD-IP-NAC	9.5 ± 1.6	40.8 ± 7.2 *
	HFD-I/R-NAC	9.3 ± 5.5	47.2 ± 10.5 *
	HFD-IP-NAC	7.9 ± 2.3	46.1 ± 8.9 *
+dP/dt_max_ (mmHg/seg)	CD-I/R	3831 ± 409	257 ± 89 *
	CD-IP	3485 ± 246	236 ± 67 *
	HFD-I/R	3892 ± 324	268 ± 72 *
	HFD-IP	3868 ± 508	365 ± 78 *
	CD-I/R-NAC	3184 ± 301	354 ± 78 *
	CD-IP-NAC	3896 ± 306	355 ± 57 *
	HFD-I/R-NAC	3546 ± 327	297 ± 56 *
	HFD-IP-NAC	3942 ± 541	372 ± 68 *
CPP (mmHg)	CD-I/R	67.1 ± 6.4	93.3 ± 10.7 *
	CD-IP	70.4 ± 3.8	87.13 ± 6.5 *
	HFD-I/R	67.1 ± 5.8	111.7 ± 13.6 *
	HFD-IP	68.0 ± 4.0	85.8 ± 6.2 *
	CD-I/R-NAC	65.1 ± 5.4	102.4 ± 9.9 *
	CD-IP-NAC	75.9 ± 4.1	99.0 ± 4.5 *
	HFD-I/R-NAC	65.5 ± 6.9	100.7 ± 14.0 *
	HFD-IP-NAC	64.8 ± 5.7	97.6 ± 6.3 *

*n* = 7, * *p* ≤ 0.05 vs. basal.

## Data Availability

The original contributions presented in this study are included in the article.

## Abbreviations

The following abbreviations are used in this manuscript:

AMI	acute myocardial infarction
CD	control diet
HFD	high-fat diet
GSH	reduced glutathione
GSSG	oxidized glutathione
IP	ischemic postconditioning
I/R	ischemia/reperfusion
NAC	*N*-acetylcysteine
ROS	reactive oxygen species
